# Oligo-Porphyran Ameliorates Neurobehavioral Deficits in Parkinsonian Mice by Regulating the PI3K/Akt/Bcl-2 Pathway

**DOI:** 10.3390/md16030082

**Published:** 2018-03-06

**Authors:** Yingjuan Liu, Lihua Geng, Jingjing Zhang, Jing Wang, Qi Zhang, Delin Duan, Quanbin Zhang

**Affiliations:** 1 Key Laboratory of Experimental Marine Biology, Institute of Oceanology, Chinese Academy of Sciences, Qingdao 266071, China; liuyingjuan829@163.com (Y.L.); genglihua13@mails.ucas.ac.cn (L.G.); jingwang@qdio.ac.cn (J.W.); zhangqi515@mails.ucas.ac.cn (Q.Z.); 2 Laboratory for Marine Biology and Biotechnology, Qingdao National Laboratory for Marine Science and Technology, Qingdao 266071, China; 3 University of the Chinese Academy of Sciences, Beijing 100049, China; 4 Pharmaceutical Department, Qingdao Eighth People’s Hospital, Qingdao 266000, China; zhangjingjing02@126.com; 5 State Key Lab of Seaweed Bioactive Substances, Qingdao 266000, China; dlduan@qdio.ac.cn

**Keywords:** oligo-porphyran, PI3K/Akt, apoptosis, Parkinson’s disease, neurobehavior

## Abstract

Parkinson’s disease (PD) is a neurodegenerative movement disorder that is caused by a selective loss of dopaminergic neurons. Current PD treatments provide symptomatic relief but do not prevent or decelerate disease progression. Previous studies have suggested that acetylated and phosphorylated porphyran, derived from *Porphyra*, produces a neuroprotective effect against 6-OHDA-induced damage. Due to its antioxidant and neuroprotective potential, this study evaluates whether oligo-porphyran (OP) could be beneficial in an experimental model of PD in mice. The drug 1-methyl-4-phenyl-1,2,3,6-tetrahydropyridine (MPTP) was intraperitoneally injected (20 mg/kg body weight) for seven days to simulate PD, followed by OP administration. We found that the behavioral deficits in spontaneous motor activity, latency to descend in a pole test, and suspension in a traction test were ameliorated, and excessive dopamine (DA) metabolism was suppressed after OP treatment. Additionally, we found that OP protected dopaminergic neurons by preventing MPTP-induced decreases in dopaminergic transporter and tyrosine hydroxylase protein levels. We speculated whether OP regulates a signaling pathway that affects the behavioral changes seen in PD mice. In this study, the PI3K/Akt/Bcl-2 pathway was detected. Our results demonstrate that OP increased the phosphorylation of PI3K/Akt/GSK-3β and inhibited the activation of caspase-3 and poly (ADP-ribose) polymerase, with changes in the Bax/Bcl-2 ratio. These results showed that OP might promote DA neuron survival in vivo by regulating the PI3K/Akt/Bcl-2 pathway, thereby ameliorating the neurobehavioral deficits in a PD mouse model and suggesting OP as a neuroprotective treatment for PD.

## 1. Introduction

Parkinson’s disease (PD) is an age-related, neurodegenerative disorder that is characterized by the gradual, progressive loss of dopaminergic neurons and decreased dopamine (DA) levels in the basal ganglia [1]. Despite decades of research, current pharmacological treatments for PD do not prevent the progressive loss of dopaminergic neurons in PD patients, and the mechanisms remain undefined [1,2]. Thus, further investigation on the molecular mechanisms of PD is indispensable to the discovery of novel therapeutic agents.

The clinical symptoms of PD include resting tremor, bradykinesia, muscle rigidity, and postural instability [3]. A previous study showed that PD motor manifestations are attributed to dopaminergic cell loss [4]. Therefore, how do motor symptoms respond to the loss of dopamine neurons? While the underlying mechanisms are not completely understood, accumulating evidence indicates a neuroprotective effect via the PI3K/Akt signaling pathway in PD models, both in vitro and in vivo [5,6]. The PI3K-Akt pathway in the brain modulates synaptic plasticity, neural survival, and cognitive function [7,8]. 

In recent years, significant attention focused on the potential of oligosaccharides to modulate neuronal function and to control neurodegenerative diseases [9,10]. Chitosan and acetylated chitosan oligosaccharides were found to have therapeutic effects on different types of neuronal disorders, including, but not limited to, Alzheimer’s disease, PD, and nerve crush injury [10]. Oligomannurarate, a novel anti-Alzheimer’s disease candidate drug derived from marine brown alga, could protect mitochondrial function by inhibiting the formation of Aβ oligomers [11]. *Porphyra*, which is an important food source worldwide, is nutritious seaweed that is abundant in proteins, polysaccharides, vitamins, and minerals [12]. Porphyran, with a linear backbone of alternating 3-linked β-d-galactose and 4-linked α-l-galactose-6-sulfate or 3,6-anhydro-α-l-galactose (3,6-AG) units [13], is a natural compound with various bioactivities, including anti-hyperlipidemia [14], anti-oxidative [15], and anti-inflammatory effects [16]. Wang et al. found that acetylated and phosphorylated porphyran antagonized the weak toxicity of 6-OHDA on MES23.5 dopaminergic cells, possessing minor neuroprotective effects that were independent of mitochondrial restoration. Oligo-porphyran (OP), a product of acid hydrolysis of porphyran, may have an effect on PD. PD motor manifestations were observed intuitively; if so, we should consider the involvement of the PI3K/Akt pathway that has been previously shown to participate in neuron survival. To test this hypothesis, the anti-PD effect of OP was evaluated in 1-methyl-4-phenyl-1,2,3,6-tetrahydropyridine (MPTP)-induced PD mice.

## 2. Results

In this study, oligo-porphyran (OP) derived from *Porphyra* was applied to investigate its neuroprotective effects in C57BL/6 mice induced by MPTP. After pretreated with MPTP for seven days, OP was administrated in the followed seven days. Interestingly, we found that OP could improve the behavioral deficits induced by MPTP. Then, we wanted to know how did OP play a part in the neuroprotection. Much research demonstrated that PI3K/Akt involved in proliferation, differentiation, and survival [17,18,19], so we speculated that oligo-porphyran might ameliorate neurobehavioral deficits in parkinsonian mice by regulating the PI3K/Akt/Bcl-2 pathway.

### 2.1. Analysis of OP

OP was prepared by acid hydrolysis of porphyran. The degree of polymerization and structure of OP was determined by high-performance liquid chromatography (HPLC) and electrospray ionization time-of-flight mass spectrometry (ESI-TOF-MS), respectively. The degree of polymerization of OP ranged from 2 to 8. OP was mainly composed of sulfated galactans and oligosaccharides with a linear backbone of alternating 3-linked β-d-galactose and 4-linked α-l-galactose-6-sulfate. The main oligosaccharide structure of OP is shown in Table 1.

### 2.2. Effects on Behavioral Patterns

**Figure 1:**
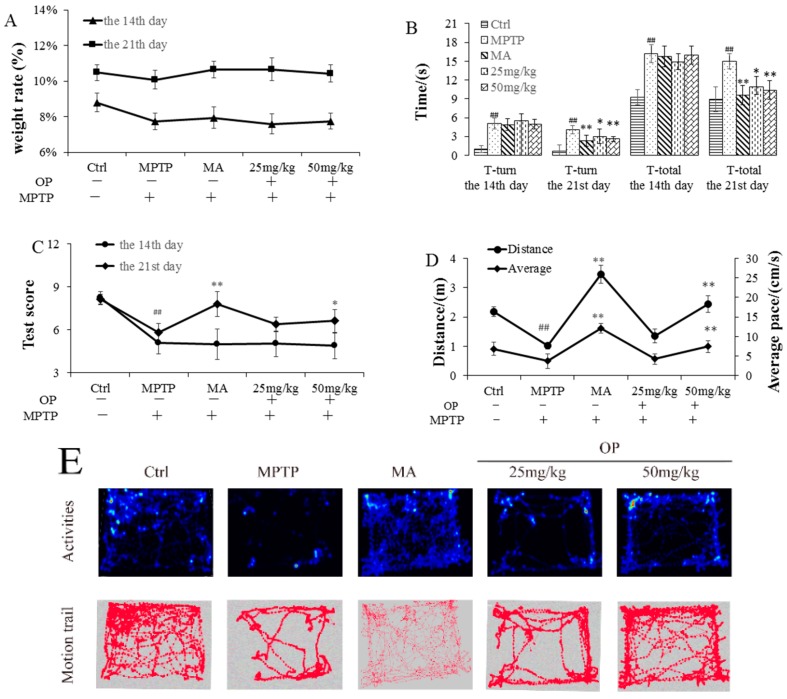
Effects of oligo-porphyran on the body weight ratio and behavioral patterns. After pretreated with 1-methyl-4-phenyl-1,2,3,6-tetrahydropyridine (MPTP) for seven days, the C57BL/6 mice were administrated with MA or different concentrations of OP for the followed seven days. (**A**): weight ratio. (**B**): pole performance at day 14 and 21; T-turn of pole test: time until the mouse turned completely downward; T-total: time until it climbed down to the floor. (**C**): traction performance at day 14 and 21. Scoring 3 for gripping the wire with both hind paws, 2 for gripping the wire with one hind paw, and 1 for not gripping the wire with either hind paw. The time of suspension was recorded. (**D**): Total movement distance and mean velocity in 5 min. (**E**): Activeness and motion trail of the mice. ^##^ *p* < 0.01 compared with the control group; * *p* < 0.05 and ** *p* < 0.01 compared with the MPTP-induced group. Data are represented as mean ± SEM, *n* = 12 mice in each group.

The pole, traction, and open field tests were performed to evaluate motor deficits in MPTP-treated mice. The body weight of each mouse was recorded during the drug treatment. No significant differences were observed between the OP-treated and MPTP-treated groups (Figure 1A). As shown in Figure 1B, after MPTP treatment, total locomotor activity time, and T-turn time were significantly prolonged; however, post-treatment with OP shortened the time needed to reach the platform (*p* < 0.01), suggesting that OP prevented MPTP-induced motor deficits. When compared with the control, the MPTP-treated mice showed decreased strength. Hind limb grip scores with hanging time on the rope were lower, but after OP treatment, we found improvements in traction test performance, suggesting that the initial lesions caused by MPTP were alleviated by OP (Figure 1C). In the open field test, as compared with the control, the MPTP-treated mice displayed a significant decrease in spontaneous motor activity (Figure 1D,E). OP significantly ameliorated these behavioral deficits that were induced by MPTP toxicity. These results suggest that OP could effectively improve behavior in the MPTP mouse model of PD. In this study, Medopa was applied as the positive control; we found that after MA treatment the behavioral patterns all were improved significantly, it might be attribute to its direct application of DA to reduce the nervon injury.

### 2.3. Effects on Quantification of DA, Norepinephrine, 5-Hydroxytryptamine, and Their Metabolites

**Figure 2:**
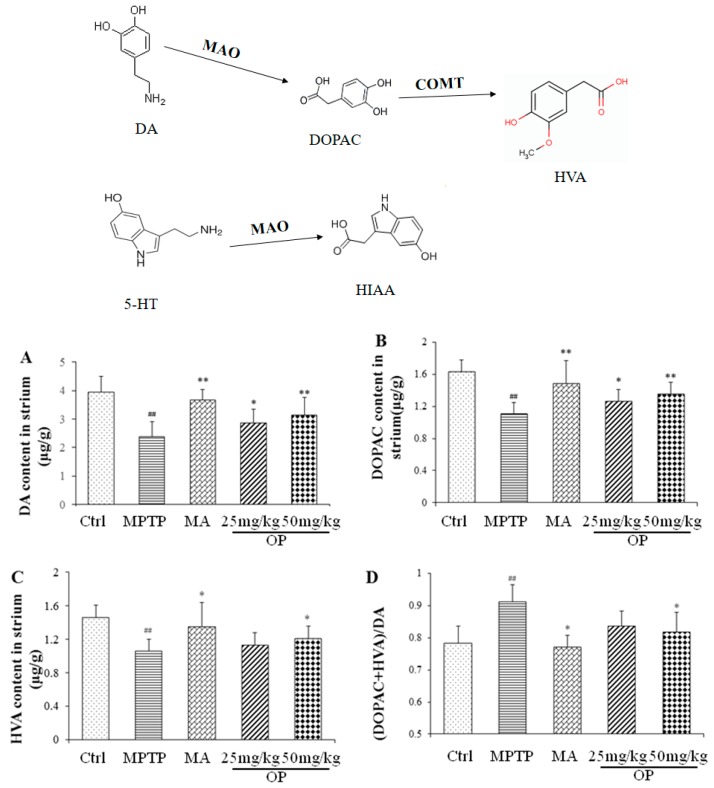

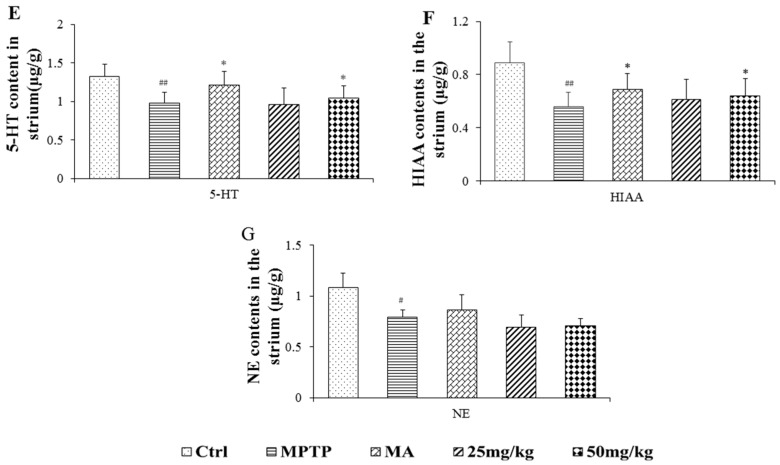
Effects of OP on the levels of dopamine (DA) (**A**), 3,4-dihydroxyphenylacetic acid (DOPAC) (**B**), HVA (**C**), MAO-dependent dopamine catabolism (**D**); 5-hydroxytryptamine (5-HT) (**E**), 5-hydroxyindoleacetic acid (HIAA) (**F**), and norepinephrine (NE) (**G**) in the striatum. After pretreated with MPTP for seven days, the C57BL/6 mice were administrated with MA or different concentrations of OP for the followed seven days. ^#^ *p* < 0.05, ^##^ *p* < 0.01 compared with the control group; * *p* < 0.05 and ** *p* < 0.01 compared with the MPTP-induced group. Data are represented as mean ± SEM, *n* = 12 mice in each group.

We observed significant effects of OP on PD mice behavior. The effects of OP on DA, norepinephrine (NE), 5-hydroxytryptamine (5-HT), and their metabolites are shown in Figure 2. The present study confirmed that administration of MPTP induced a marked decrease in DA levels and its metabolites when compared to those in the control group (Figure 2A–C, *p* < 0.05). However, administration of OP and MA significantly attenuated the decrease in DA levels and 3,4-dihydroxyphenylacetic acid (DOPAC, a DA metabolite) (Figure 2A,C, *p* < 0.01). Furthermore, the (DOPAC + homovanillic acid (HVA)): DA ratio, indicating monoamine oxidase (MAO)-dependent dopamine catabolism, was enhanced by MPTP; but, intraperitoneal treatment with OP strongly depressed the MPTP-evoked acceleration of MAO-dependent dopamine catabolism (Figure 2D), and the effect preceded the MA treatment, suggesting that MA failed to inhibit the neurons injury, and OP presented a promising potential. In addition, the levels of 5-HT and its metabolite, 5-hydroxyindoleacetic acid (HIAA), were significantly enhanced by MPTP, as previously reported [20]. The degree of enhancement decreased with increasing concentrations of OP (25 and 50 mg/kg; Figure 2E,F). We observed no significant changes in NE levels (Figure 2G).

### 2.4. Effects on Expression of Extracellular Signal-Regulated Protein Kinases 1 and 2, Dopamine Receptor D2, Dopamine Receptor D2, and Dopamine Transporter

**Figure 3:**
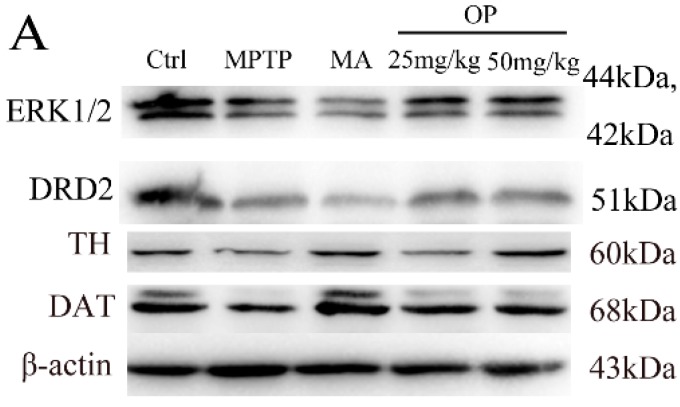

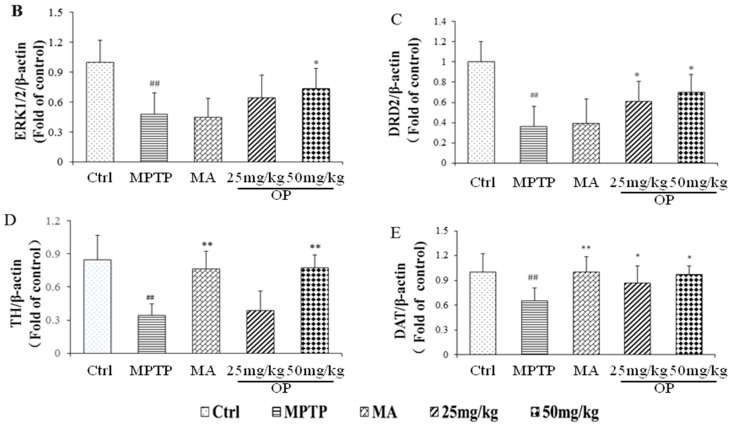
Effects of OP on the levels of expression of extracellular signal-regulated protein kinases 1 and 2 (ERK1/2), dopamine receptor D2 (DRD2), tyrosine hydroxylase (TH), and dopamine transporter (DAT) in the striatum. After pretreated with MPTP for seven days, the C57BL/6 mice were administrated with MA or different concentrations of OP for the followed seven days. (**A**): Original bands of ERK1/2, DRD2, TH, DAT, and β-actin. (**B**): Quantitative density of ERK1/2. (**C**): Quantitative density of DRD2. (**D**): Quantitative density of TH. (**E**): Quantitative density of DAT. ^##^ *p* < 0.01 compared with the control group; * *p* < 0.05 and ** *p* < 0.01 compared with the MPTP-induced group. Data are represented as mean ± SEM, *n* = 12 mice in each group.

In agreement with previous reports, MPTP induced a severe reduction in tyrosine hydroxylase (TH)-positive cells. However, OP treatment (50 mg/kg) significantly rescued degeneration, an effect comparable to that of Madopar, a clinical anti-PD drug. While MPTP treatment caused a marked reduction of dopamine transporter (DAT) and dopamine receptor D2 (DRD2), OP treatment resulted in a remarkable recovery (*p* < 0.05). In accordance with the above results, OP treatment markedly restored the decrease in phosphorylation of extracellular signal-regulated protein kinases 1 and 2 (ERK1/2) induced by MPTP (*p* < 0.05, Figure 3B).

### 2.5. Effects on PI3K, Akt, and GSK 3β Expression

**Figure 4:**
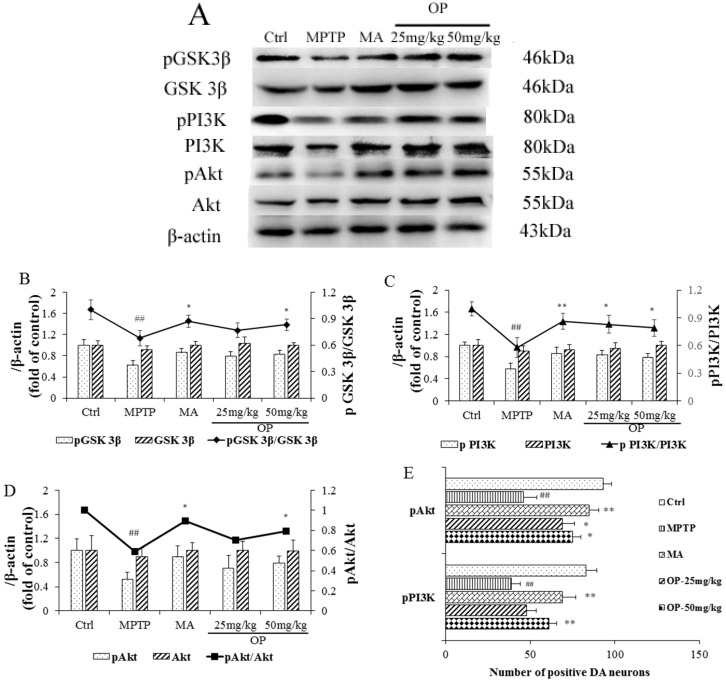
Effects on the phosphorylation of phosphoinositide 3-kinase (PI3K)/protein kinase B (Akt), and glycogen synthase kinase 3 (GSK 3β). After pretreated with MPTP for seven days, the C57BL/6 mice were administrated with MA or different concentrations of OP for the followed seven days. (**A**): Original bands of pPI3K, PI3K, pAkt, Akt, pGsk 3β, Gsk 3β, and β-actin. (**B**): Quantitative density of pGsk 3β, Gsk 3β, and the ratio of pGsk 3β/Gsk 3β. (**C**): Quantitative density of pPI3K, PI3K, and the ratio of pPI3K/PI3K. (**D**): Quantiative density of pAkt, Akt, and the ratio of pAkt/Akt. (**E**): Examation about whether OP affected GSK-3β (Ser9) phosphorylation. ^##^ *p* < 0.01 compared with the control group; * *p* < 0.05 and ** *p* < 0.01 compared with the MPTP-induced group. Data are represented as mean ± SEM, *n* = 12 mice in each group.

As shown above, OP improved motor deficits in PD mice and inhibited the excessive consumption of DA. We speculated that OP might regulate some signal pathway to accelerate neuronal survival. We assessed the possible involvement of the intracellular PI3K/Akt signaling pathway in MPTP-treated mice. Western blot analysis revealed that phosphorylation of phosphoinositide 3-kinase (PI3K), protein kinase B (Akt), and glycogen synthase kinase 3 (GSK 3β) were markedly decreased in MPTP-induced mice when compared to the control (0.57-, 0.59-, 0.68-fold, *p* < 0.01), suggesting the inhibition of the PI3K/Akt signaling pathway. However, mice from the OP group exhibited a significant increase in the phosphorylation of PI3K (Figure 4C). Furthermore, OP (50 mg/kg) administration significantly increased Akt (Ser473) phosphorylation (Figure 4D, 1.35-fold compared with MPTP, *p* < 0.05), the effect was on a par with that of MA. We performed immunohistochemical analysis of PI3K and Akt to elucidate the molecular mechanisms underlying the effects of OP. We found that OP increased the positive neurons of phosphorylation in PI3K and Akt. We next examined whether OP affected GSK-3β (Ser9) phosphorylation. When compared to the MPTP-treated group, OP had no significant effects on GSK-3β levels, but increased GSK-3β phosphorylation (Figure 4B, 1.22-fold, *p* < 0.05). These results suggest that OP regulates the PI3K/Akt pathway providing neuroprotective effect.

### 2.6. Effects on Bax/Bcl-2, CytC, Poly ADP Ribose Polymerase, and Cleaved Caspase-3 Levels

**Figure 5:**
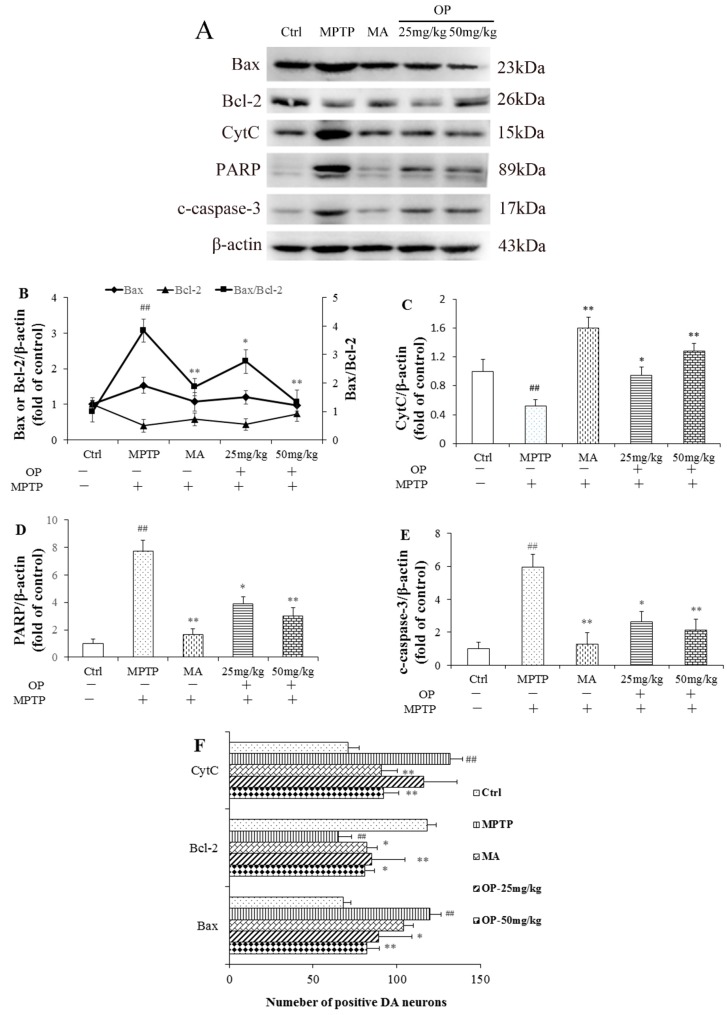
Effects on apoptosis-related protein expression, poly ADP ribose polymerase (PARP), and caspase-3 activity in vivo. After pretreated with MPTP for seven days, the C57BL/6 mice were administrated with MA or different concentrations of OP for the followed 7 days. (**A**): Original bands of cytochrome c (CytC), cleaved caspased-3, B-cell lymphoma 2 (Bcl-2), BCL2 associated X (Bax), PARP, and β-actin. (**B**): Quantitative density of Bcl-2, Bax, and Bcl-2/Bax ratio. (**C**): Quantitative density of CytC. (**D**): Quantitative density of PARP. (**E**): Quantitative density of cleaved caspase-3. (**F**): Bax, Bcl-2, and CytC immunostaing. ^##^ *p* < 0.01 compared with the control group; * *p* < 0.05 and ** *p* < 0.01 compared with the MPTP-induced group. Data are represented as mean ± SEM, *n* = 12 mice in each group.

The mitochondrial apoptosis pathway plays an important role in the apoptosis of dopaminergic neurons [6]. As shown in Figure 5, MPTP increased the expression of BCL2 associated X (Bax) and decreased the expression of B-cell lymphoma 2 (Bcl-2), resulting in an upregulation of the Bax/Bcl-2 ratio compared with that in the control group; however, OP (25, 50 mg/kg) decreased the ratio significantly (Figure 5B). Figure 5C showed that when compared to the control group, MPTP induced the release of cytochrome c (CytC), whereas post-treatment with OP prevented CytC release. Similar results could be observed in the expression of poly ADP ribose polymerase (PARP) and cleaved caspase-3, in that OP inhibited the increase of PARP and the activated form of caspase-3 after MPTP treatment. Immunohistochemical analysis for Bax, Bcl-2, and CytC also showed that OP reduced the number of immunopositive neurons for pro-apoptotic proteins, Bax and CytC, and increased the number of immunopositive neurons for anti-apoptosis protein, Bcl-2 (Figure 5F). These results suggest that OP could inhibit mitochondrial apoptosis, thereby preventing cell injury. However, MA showed slight effect on the inhibition of apoptosis.

### 2.7. Effects on Nerve Growth Factor and Tropomyosin Receptor Kinase A Levels

**Figure 6:**
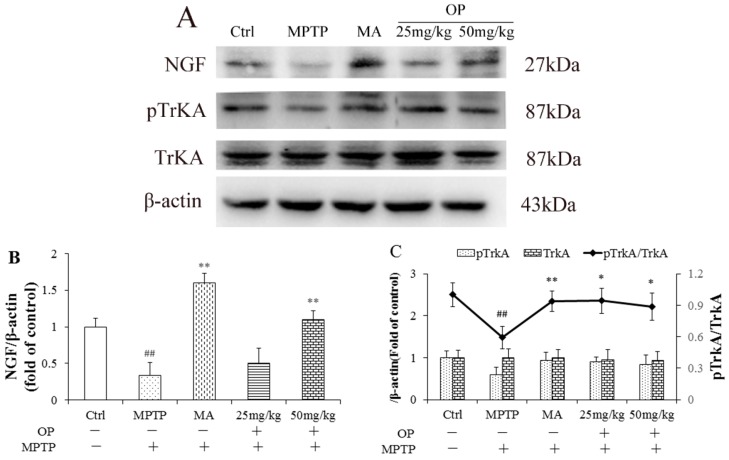
Effects of OP on nerve growth factor (NGF) and tropomyosin receptor kinase A (TrkA) expression. After pretreated with MPTP for 7 days, the C57BL/6 mice were administrated with MA or different concentrations of OP for the followed 7 days. (**A**) Original bands of NGF, pTrkA, TrkA, and *β*-actin. (**B**) Quantitative density of NGF. (**C**) Quantitative density of pTrkA, TrkA, and pTrkA/TrkA ratio. ^##^ *p* < 0.01 compared with the control group; * *p* < 0.05 and ** *p* < 0.01 compared with the MPTP-induced group. Data are represented as mean ± SEM, *n* = 12 mice in each group.

Subsequently, we detected the upstream nerve growth factor (NGF) and tropomyosin receptor kinase A (TrkA) of the signalling pathway. Western blot analyses show MPTP attenuated the level of NGF and the phosphorylation of TrkA, whereas OP, at a dose of 50 mg/kg/day, effectively increased TrkA and NGF phosphorylation in the striatum (Figure 6), presenting the similar effects to MA.

### 2.8. Effects on Dopaminergic Neuronal Loss

**Figure 7:**
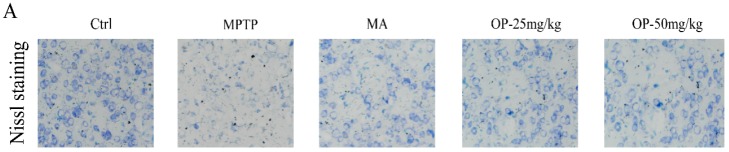

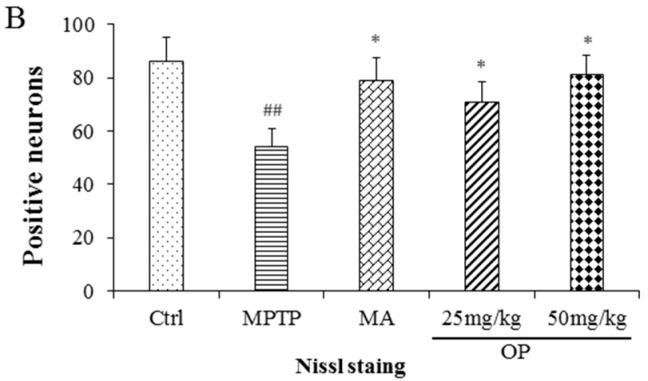
Effects of OP on dopaminergic neuronal loss. (**A**) Nissl staining. (**B**) Quantitative Nissl positive neurons. Data are represented as mean ± SEM. ^##^ *p* < 0.01 compared with the control group; * *p* < 0.05 compared with the MPTP-induced group. Data are represented as mean ± SEM, *n* = 12 mice in each group.

To observe the neuroprotective effects of OP, striatal sections were Nissl-stained. The MPTP-treated group showed significantly fewer positive cells than did the control group. However, pretreatment with OP at 25 and 50 mg/kg significantly reduced this loss. The results showed that OP protected against MPTP-induced dopaminergic neuronal loss (Figure 7). 

## 3. Discussion

Current PD treatment involves the treatment of symptoms, such as movement disorders. For example, administration of L-DOPA elevates dopamine levels, thereby enhancing movement [21]. However, no therapies that are capable of stopping or slowing the progression of PD are available, and ultimately the patient succumbs to the disease, usually within 10 years of diagnosis [8]. Thus, it is necessary to find new therapies and drug treatments for PD. Natural products and derivates are considered relatively safe, with limited side effects, and will become an important source for clinical medications of PD in the future [10,22]. Here, we selected OP to understand its influence on a PD mouse model.

Motor function is usually applied as the evaluation index of PD [3]. Our study showed that OP effectively increased the time on a traction wire, elevated the grasping force and reaction velocity on a climbing pole, and improved behavior in an alien open field, when compared with MPTP. These results suggest that OP is potential agent to counteract PD. Moreover, it was found that its effect was closely associated with the protection of nigrostriatal dopaminergic neurons against MPTP-induced neurotoxicity. We observed that OP could attenuate the reduction in DA and 5-HT in the striatum of MPTP-treated mice. Furthermore, OP strongly inhibited the acceleration of MAO-dependent DA catabolism, as indicated by a reduction in the (DOPAC + HVA)/DA ratio. It is well known that the enzymatic catabolism of DA results in the production of DOPAC and hydrogen peroxide, which could be translated into highly toxic hydroxyl radicals. 

To further demonstrate the effects of OP on PD, we conducted Nissl staining and western blot analysis for TH, DAT, and DRD2. The early loss of DAT, followed by a reduction in TH protein levels, is thought to contribute to DA deficiency [23,24]. Our study found that OP improved the expression of TH and DAT in comparison to the MPTP-treated group. OP could also increase DRD2 levels, inhibiting the over-metabolism of DA, as demonstrated by the fact that OP increased the number of Nissl-stained cells when compared to the MPTP treated group. All of these results suggest that OP could be a promising agent for PD treatment.

Several studies have suggested that multiple intricate cell signaling cascades [25], including PI3K/Akt, Wnt/β-catenin, are involved in neuron proliferation, differentiation, and survival [7,8,17]. Herein, we speculate that OP might protect neurons by regulating the recruitment of the PI3K/Akt signaling pathway, at the receptor or kinase level, which increases the expression of neuroprotective and neuromodulatory proteins. To explore the upstream signaling pathway that is involved in the neuroprotective effects, OP, NGF, and TrkA were investigated simultaneously. In agreement with a previous study of mice treated with 1-methyl-4-phenylpyridinium ion (MPP+, a toxic MPTP metabolite) alone, TrkA phosphorylation decreased significantly, as well as NGF levels, as seen in our experiments. Likewise, phosphorylated ERK1/2 levels decreased markedly in MPTP-treated mice. However, OP treatment attenuated the downregulation of TrkA and ERK1/2 phosphorylation and increased NGF levels that were caused by MPTP.

Basal levels of PI3K and Akt and their phosphorylation were evaluated following OP treatment and were not altered by OP in the striatum. Notably, levels of phosphorylated PI3K and Akt were significantly upregulated when compared with those in the MPTP-treated group. Glycogen synthase kinase-3β, a downstream target of Akt, exerts a negative effect on neurogenesis and could be inhibited by activated Akt through inducing its phosphorylation [25]. OP-activated Akt and GSK3β were then suppressed. These results provide robust clues that OP reduces death of dopaminergic neurons induced by MPTP by acting on the PI3K/Akt signaling pathway.

Several pro-apoptotic proteins, including Bcl-2-associated death promoter, Bax, and caspase-9, are the downstream targets of the PI3K/Akt pathway [26,27]. Activated Akt, consequently, inactivates apoptins, thereby promoting cell survival. The balance of pro-apoptotic and anti-apoptotic proteins in the Bcl-2 family plays a central role in the regulation of caspase activation [28]. Numerous studies have shown that MPTP disequilibrates the ratio of Bcl-2/Bax in dopaminergic neurons [29]. In our experiments, MPTP significantly decreased the Bcl-2/Bax ratio in the striatum of mice. OP treatment reversed the effect on the ratio of Bcl-2/Bax, and this result was in keeping with the fact that OP inhibited the activation of caspase-3 and PARP as induced by MPTP. Cell apoptosis was proven through interleukin 1 beta (IL-1β) stimulation of active caspase-3 and cleaved PARP expression. Therefore, the results suggest that OP prevents mitochondrial dysfunction-induced apoptosis of dopaminergic neurons.

## 4. Materials and Methods

### 4.1. Chemicals

Madopar, which is a combination of levodopa and benserazide, was obtained from Shanghai Roche Pharmaceuticals Ltd. (Shanghai, China). Dopamine, DOPAC, HVA, 5-HT, NE, HIAA, and MPTP were purchased from Sigma-Aldrich (St. Louis, MO, USA). A bicinchoninic acid (BCA) protein determination kit was obtained from CoWin Biosciences, Beijing, China. Diaminobenzidine (DAB) staining, and Nissl staining kits were purchased from Beyotime (Nanjing, China). The Polyvinylidene fluoride (PVDF) transfer membrane was purchased from Millipore Corp. (Bedford, MA, USA). Primary antibodies were obtained from Affinity Biosciences, Cell Signal Transduction, Cincinnati, OH, USA. Table 2 shows the primary antibody specifications and dilutions.

### 4.2. Preparation and Analysis of OP

The porphyran used for hydrolysis was prepared from *Porphyra capensis*, as described previously [12]. In brief, the alga was autoclaved for 3 h at 120 °C and were successively filtered through gauze and siliceous earth as filtering aids. The supernatant was dialyzed against running tap water overnight then against distilled water for 12 h. The solution was then lyophilized to obtain dry porphyran.

A 2.5% *w*/*v* porphyran solution was added to H_2_SO_4_ up to a final concentration of 0.5 mol/L and incubated for 3 h at 80 °C. After acid hydrolysis, BaCl_2_ was added to neutralize the solution to a pH 7.0, and then centrifuged to remove sediments. The supernatant was lyophilized to obtain OP [30].

### 4.3. Animal Treatment

Male C57BL6 mice (mean weight = 22 g; mean age = 8 weeks) were used in the present study. The mice received care in compliance with the Principles of Laboratory Animal Care as developed by the National Society for Medical Research, which was approved by the institutional Animal Care and Ethics Committee. The mice were individually housed in cages under controlled conditions (humidity, temperature, 12 h light/dark cycle) by skilled veterinarians and technicians at the Zhonghao animal center, with free access to food and tap water. 

**Figure 8:**
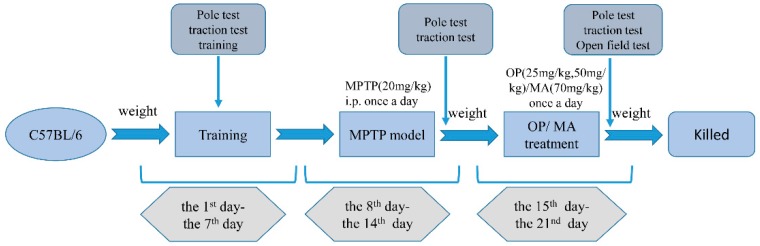
Schematic diagram of experimental procedure.

The mice were randomly separated into five groups, with 12 mice in each group: (1) control, (2) MPTP only, (3) MPTP + Madopar (70 mg/kg), (4) MPTP + OP (25 mg/kg), and (5) MPTP + OP (50 mg/kg). For the first seven days, the mice adapted to the environment and trained on a pole test and traction test. In the following seven days, MPTP hydrochloride was injected intraperitoneally at a dose of 20 mg/kg each day, while the control group received the same volume of saline. OP or Madopar were then administered intraperitoneally for another seven consecutive days, while the control and MPTP groups received equivalent volumes of saline. Locomotion, coordination, and balance skills were evaluated after administration was complete. The schematic diagram of experimental procedure was shown in Figure 8.

### 4.4. Behavioral Evaluation

A trained observer carried out all behavioral experiments, blind to treatment, between 10:00 and 15:00 in a weakly-lit and quiet environment. Behavioral assessments, including the pole test, traction test, and open-field tests, are frequently used to determine parkinsonism-related outcomes.

#### 4.4.1. Open Field Test

We tested locomotion by placing each mouse in an open arena (44 × 44 × 32 cm^3^) and recording its activity for 5 min with weak light (40 W) illumination. A video camera, which was placed over the arena, was used to detect the position of the mouse. 

EthoVision video tracking system software (The Noldus Community, Wageningen, The Netherlands) was used for data collection and processing, providing measures of total distance travelled, mean velocity (*V_mean_*), and activity. The average time of three tests was calculated for statistical analyses.

#### 4.4.2. Pole Test

Coordination was assessed using a pole test, following a slightly modified version of a previously described protocol [31]. A pole, 50 cm high, 1 cm in diameter, was wrapped in gauze to prevent slipping with a 1 cm diameter ball glued on top. The mice were placed head upward on the top of the pole, and the total time (T-total) taken to climb down the pole, along with the time at which the mouse turned downward (T-turn), were measured. The average time for three tests was calculated for statistical analyses.

#### 4.4.3. Traction Test

Limb impairment was assessed by a traction test, as described previously [31]. Briefly, the mice were hung from a horizontal wire by their forepaws. A mouse scored three points if it grasped the wire with both hind paws, two points if it grasped the wire with one hind paw, and one point if it did not grasp the wire with either hind paw. The suspension times were recorded. A scored of 0 was given if the time was 0–4 s, one point for times between 5–9 s, two points for times between 10–14 s, three points for times between 15–19 s, four points for times between 20–24 s, five points for times between 25–29 s, and six points if the time was ≥30 s. The average time for three tests was calculated for statistical analyses.

### 4.5. Collection of Brain Tissue

After the behavioral experiments, seven mice in each group were anesthetized with 10% *v*/*v* chloral hydrate, followed by dissection on ice to collect the striatum. The remaining mice were perfused intracardially with phosphate buffer solution (PBS), followed by 4% *v*/*v* paraformaldehyde (PFA). Brains were dissected and fixed in 4% *v*/*v* PFA at 4 °C overnight and were then dehydrated successively with 20% and 30% sucrose solution (*w*/*v*) prepared in PBS, embedded in optimum cutting temperature (O.C.T., Sakura Finetek, USA, Inc., Torrance, CA, USA) compound, and cut into 20 μm-thick sections on a Leica microtome (Leica Biosystems Nussloch GmbH, Heidelberger, Germany).

### 4.6. HPLC Analysis of DA, 5-HT, NE, and Their Metabolites

Striatal tissue was processed and stored at −80 °C. Dopamine, 5-HT, NE, and their metabolites, DOPAC, HVA, and 5-HIAA were simultaneously quantified using an optimized reverse-phase high-performance liquid chromatography (RP-HPLC) apparatus (YMC-Pack ODS-AQ, 100 × 2.1 mm^2^, S-3 μm, YMC Europe GmbH, Dinslaken, Germany) with ion-pairing (1-octanesulphonic acid; OSA) and amperometric electrochemical detection, as previously published [32,33]. The striatal tissues were homogenized in 300 μL of 0.2 M perchloric acid. After 60 min in an ice bath, the samples were centrifuged at 12,000× *g* for 20 min at 4 °C. The supernatant was added to a solution of 0.3 M potassium dihydrogen phosphate, 0.02 M potassium citrate, and 0.002 M Na_2_EDTA. After another 60 min in an ice bath, the mixture was centrifuged at 12,000 × *g* for 20 min at 4 °C. The supernatant was filtered through a 0.22 μm Millipore filter and was analyzed by HPLC. The mobile phase consisted of 1.7 mM 1-octanesulfonic acid sodium salt, 1.0 mM Na_2_EDTA × 2 H_2_O, 8.0 mM NaCl, 100 mM NaH_2_PO_4_ × 2 H_2_O (pH 3.80), mixed with 9.3% *v*/*v* acetonitrile, and was delivered with a flow rate of 0.4 mL/min. The mobile phase was constituted in 0.125 M sodium citrate buffer containing 20% *v*/*v* methanol, 0.1 mM Na_2_EDTA, and 0.5 mM 1-octanesulfonic acid sodium salt adjusted to a pH 4.3.

### 4.7. Western Blotting Analysis

Tissues stored at −80 °C were homogenized in tissue lysis reagent supplemented with a protease inhibitor. The lysate was centrifuged at 12,000 rpm for 15 min at 4 °C. Protein concentrations were detected using a BCA kit. The extracted proteins from each sample were separated by sodium dodecyl sulfate-polyacrylamide gel electrophoresis (10% or 12%) and transferred onto PVDF membranes. The membranes were blocked with 5% *v*/*v* bovine serum albumin solution at room temperature for 1 h and were incubated with their respective primary rabbit antibodies to NGF, TH, DAT, DRD2, PARP, ERK1/2, pTrk A, Trk A, pAkt, Akt, pGSK 3β, GSK 3β, pPI3K, PI3K, Bax, Bcl-2, cleaved-caspase-3, CytC, and β-actin overnight at 4 °C. After washing with 1 × PBST (PBS containing 0.1% *v*/*v* Tween 20), the membranes were incubated with their respective secondary antibodies. β-Actin was used as an internal reference (1:2000, Affinity Biosciences, Cincinnati, OH, USA). The tests were repeated 3 times. The protein bands were visualized using the ECL-prime kit (Affinity Biosciences, Cincinnati, OH, USA) and quantified with Image Lab software (Bio-Rad Laboratories, Inc., Hercules, CA, USA).

### 4.8. Immunohistochemistry

After inactivation of endogenous peroxidases with 3% *v*/*v* H_2_O_2_ for 10 min, the sections were permeabilized in 0.3% *v*/*v* TritonX-100 for 20 min and was blocked with 10% *v*/*v* goat serum for an hour. Subsequently, they were incubated with primary antibodies against pPI3K (1:200), pAkt (1:200), CytC (1:200), Bax (1:200), and Bcl-2 (1:200) at 4 °C overnight, and then incubated with secondary antibodies (1:500) conjugated with streptavidin-labeled peroxidase, followed by DAB coloration. The sections were dehydrated in increasing alcohol concentrations (50%, 70%, 80%, 90%, 100% *v*/*v*) and made transparent with dimethylbenzene twice. Immunopositive neurons were quantified in six sections throughout the entire rostrocaudal extent of the striatum. Images were obtained with an Olympus Fluoview FV1000 (Olympus Corporation, Beijing, China) and the relative expression levels of the respective molecules in the striatum were analyzed using Image J 8.0 (Media Cybernetics, Rockville, MD, USA).

### 4.9. Statistical Analysis

Data analysis was performed using SPSS Version 17.0 (SPSS Inc., Chicago, IL, USA). In order to compare the mean differences among the groups, one-way analysis of variance was used. The results are shown as the mean ± standard deviation, and *p* ≤ 0.05 was considered to be statistically significant.

## 5. Conclusions

In this study, OP treatment attenuated the behavioral deficits associated with MPTP-induced lesions and improved motor behavior by mitigating apoptosis and preventing loss of dopaminergic neurons. Its beneficial effect is partly reliant on the PI3K/Akt/Bcl-2 signaling cascade. These results suggested that OP might contribute to neuroprotective activity by regulating the PI3K/Akt/Bcl-2 signaling pathway to improve motor behavior.

## Figures and Tables

**Table 1:** The main oligosaccharide structure of Oligo-porphyran (OP) analyzed by electrospray ionization time-of-flight mass spectrometry (ESI-TOF-MS).

Peak	Structure
421.07, *z* = 1	[Gal_2_-SO_3_H-H]^−^
583.12, *z* = 1	[Gal_3_-SO_3_H-H]^−^
412.06, *z* = 2	[Gal_4_-(SO_3_H)_2_-2H]^2−^
493.09, *z* = 2	[Gal_5_-(SO_3_H)_2_-2H]^2−^
409.06, *z* = 3	[Gal_6_-(SO_3_H)_3_-3H]^3−^
463.08, *z* = 3	[Gal_7_-(SO_3_H)_3_-3H]^3−^
517.09, *z* = 3	[Gal_8_-(SO_3_H)_3_-3H]^3−^

**Table 2:** Antibody information.

Antibody	Host	Application	Source	Dilutions
NGF	Rabbit	WB	Affinity Biosciences	1:1000
TH	Rabbit	WB	Affinity Biosciences	1:1000
DRD2	Rabbit	WB	Affinity Biosciences	1:1000
PARP	Rabbit	WB	Affinity Biosciences	1:1000
ERK1/2	Rabbit	WB	Affinity Biosciences	1:1000
pTrkA	Rabbit	WB/IHC	Affinity Biosciences	1:1000
TrkA	Rabbit	WB	Affinity Biosciences	1:1000
pAkt	Rabbit	WB/IHC	Affinity Biosciences	1:1000/1:200
Akt	Rabbit	WB/IHC	Affinity Biosciences	1:1000
pGSK 3β	Rabbit	WB/IHC	Affinity Biosciences	1:1000
GSK 3β	Rabbit	WB/IHC	Affinity Biosciences	1:1000
pPI3K	Rabbit	WB/IHC	Affinity Biosciences	1:1000/1:200
PI3K	Rabbit	WB/IHC	Affinity Biosciences	1:1000
Bax	Rabbit	WB/IHC	Affinity Biosciences	1:1000/1:200
Bcl-2	Mouse	WB/IHC	Affinity Biosciences	1:1000/1:200
c-caspase-3	Rabbit	WB	Abcam	1:1000
CytC	Rabbit	WB/IHC	Affinity Biosciences	1:1000/1:200
β-actin	Rabbit	WB	Affinity Biosciences	1:2000

WB: Western blot; IHC: Immunohistochemistry.
